# Supplementary material for: Causal Relationship between Aging and Anorexia Nervosa: A White-Matter-Microstructure-Mediated Mendelian Randomization Analysis
Source: Biomedicines. 2024 Aug 16;12(8):1874. doi: 10.3390/biomedicines12081874 (PMC11351342; doi:10.3390/biomedicines12081874)
Supplement: Supplementary file 1 [file biomedicines-12-01874-s001.zip › Supplementary Figure/Figure S9/Leaveoneout plot of MR analysis for ubm-b-1805 to AN.pdf]

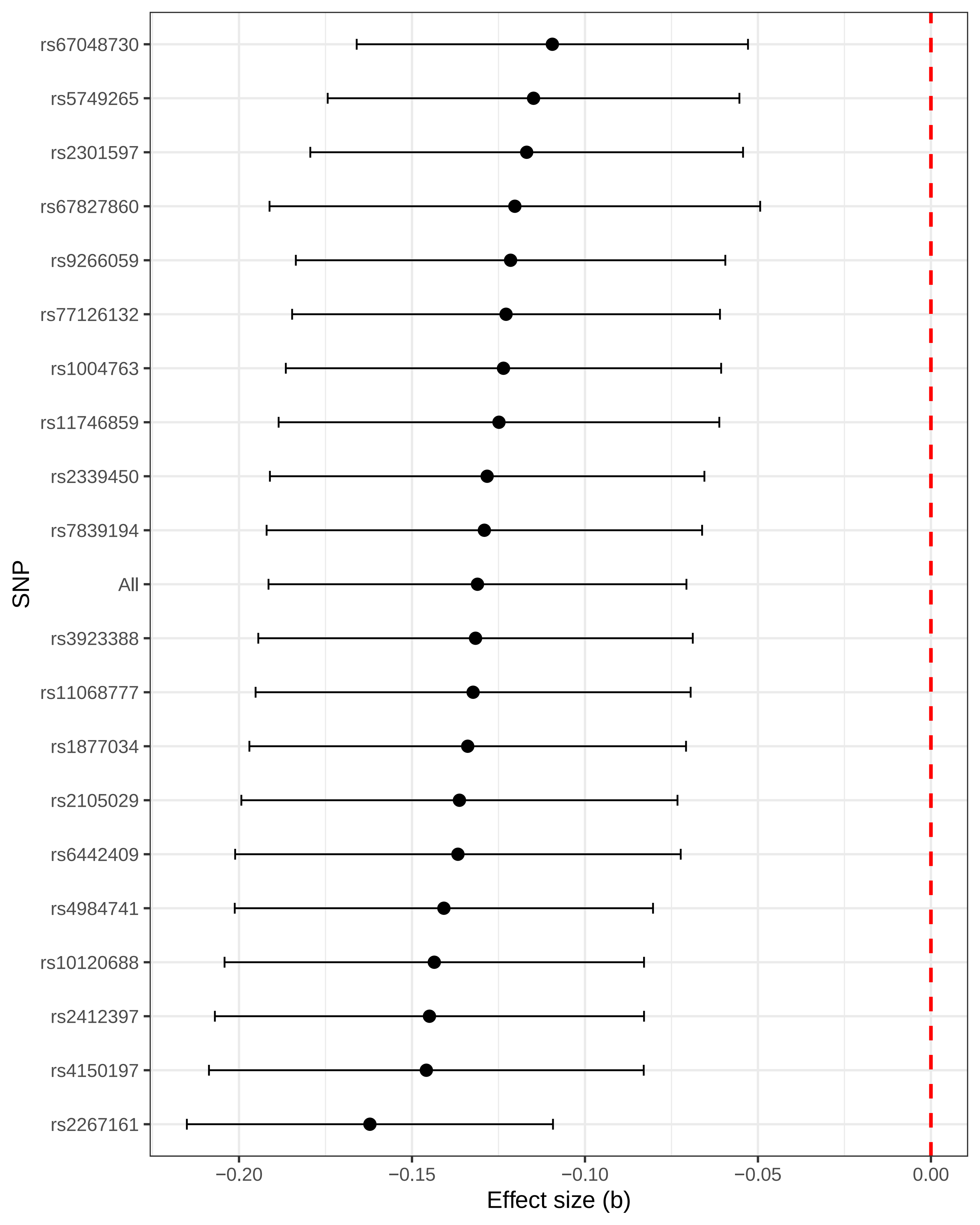
rs67048730
rs5749265
rs2301597
rs67827860
rs9266059
rs77126132
rs1004763
rs11746859
rs2339450
rs7839194
All
rs3923388
rs11068777
rs1877034
rs2105029
rs6442409
rs4984741
rs10120688
rs2412397
rs4150197
rs2267161
SNP
−0.20
−0.15
−0.10
−0.05
0.00
Effect size (b)
